# Prevalence and Impact of Diabetes Mellitus in Gout: Analysis of a Nationwide Dataset of 192,062 Hospitalizations

**DOI:** 10.3390/jcm15051925

**Published:** 2026-03-03

**Authors:** Patricia Mora-Vázquez, Fernando Borrás, Eugenio De Miguel, Antonio Picó, Mariano Andrés

**Affiliations:** 1 Departments of Clinical Medicine and of Statistics, Mathematics and Informatics, Miguel Hernandez University of Elche, 03202 Alicante, Spain; patricia.mora03@goumh.umh.es (P.M.-V.); f.borras@umh.es (F.B.); antonio.pico@umh.es (A.P.); 2 Department of Rheumatology, Hospital Universitario La Paz, 28046 Madrid, Spain; eugenio.demiguel@gmail.com; 3 Departments of Endocrinology and Nutrition and of Rheumatology, Dr. Balmis General University Hospital, 03010 Alicante, Spain; 4 Alicante Institute for Health and Biomedical Research (ISABIAL), 03010 Alicante, Spain

**Keywords:** gout, diabetes mellitus, comorbidities, recurrent admission, cardiovascular disease, infections

## Abstract

**Objectives**: The variable clustering of comorbidities in gout, including diabetes mellitus (DM), remains poorly understood. We analyzed the frequency and impact of DM in a nationwide Spanish hospitalized population with gout. **Methods**: Observational, multicenter, longitudinal study assessing 192,062 hospitalizations with gout in Spain from 2005 to 2015 (Minimal Basic Data Set, ICD-9 coding)**.** We estimated the prevalence of DM with 95% confidence intervals (CIs), stratified by DM type and related complications. A logistic regression analysis identified characteristics of patients with both gout and DM. We also matched recurrent admissions in the first tercile (2005–2008) to assess cardiovascular, renal, infectious, and thromboembolic comorbidities in the subsequent terciles (2009–2012 and 2012–2015). **Results**: DM was identified in 27.72% of the hospital-based gout population, predominantly type 2 DM, with 19.76% having complications. DM was associated with older age, female gender, and conditions such as dyslipidemia, obesity, cardiovascular and kidney diseases, liver disease, obstructive pulmonary disease, urinary tract infections, and dementia. In contrast, non-DM patients showed higher rates of venous thromboembolism and other rheumatic diseases. Readmissions were significantly more common in DM patients, who experienced +10% more cardiovascular and renal issues, similar infections, and fewer venous thromboembolism cases. **Conclusions**: DM is prevalent in gout and associated with older patients, women, and a particular comorbidity profile. The presence of DM increases the risks of readmission and the development of cardiovascular and renal diseases. Fewer venous thromboses were noted. Thus, diagnosing and managing DM in patients with gout is likely a more pressing issue.

## 1. Introduction

Gout is the most prevalent inflammatory arthritis worldwide, affecting up to 5% of adults [1]. The disease is caused by the crystallization of monosodium urate (MSU) in joints and surrounding tissues, where an inflammatory response to the crystals from the innate immune system occurs [2]. Swollen and painful joints in the lower limbs characterize acute flares. However, during intercritical periods, individuals still report pain, disability, and impaired quality of life [3]. If left untreated or poorly managed, the buildup of MSU crystals can significantly increase, leading to the formation of tophi—subcutaneous nodules filled with well-organized crystals. Tophi growth and accompanying inflammation result in erosive joint damage and related complications. Sustained normalization of serum urate levels can dissolve MSU crystals, leading to the disappearance of flares and tophi [4].

Most patients with gout present with comorbidities such as obesity, diabetes mellitus (DM), dyslipidemia, hypertension, or fatty liver disease. The prevalence of those comorbidities overpasses the general population [5]. Gout patients also have an increased risk of cardiovascular and renal diseases [6,7] and higher mortality rates [8,9], but varying on whether the management follows a urate treat-to-target strategy [10]. The range of comorbidities among individuals with gout varies significantly, and some clustering patterns have been described [11,12,13,14,15]. Although the models differ across studies and lack clear boundaries, the reported clusters include hyperlipidemia and metabolic syndrome (MS), obesity, hypertension, renal and cardiovascular diseases associated with the use of diuretics, as well as cases of isolated gout in young people with few accompanying comorbidities. These association studies are cross-sectional, and their implications for the care of individuals with gout still need to be established.

DM is commonly observed in patients with gout. Hyperuricemia is closely linked to MS and DM. Hyperuricemia may favor fatty liver, insulin resistance, obesity, and MS by systemic inflammation and oxidative stress [16,17], as well as a result of fructose consumption [18]—which is also associated with increased urate levels [19]. Additionally, hyperinsulinemia enhances urate reabsorption in the proximal convoluted tubule of the kidney, leading to decreased urinary urate excretion and increased serum urate levels [20]. In cluster analyses, DM is commonly associated with various metabolic diseases in gout populations, including dyslipidemia, MS, and hypertension [14]. Studies have shown differing associations of DM with cardiovascular and renal diseases [11] or with liver diseases and alcohol consumption [12]. Also, the comorbidity profile in DM seems to vary by sex, with women showing a higher risk of subsequent cardiovascular diseases [13]. Nevertheless, the shifting clustering of comorbidities in gout, including DM, remains poorly understood and appears to be influenced by multiple factors [21].

This study will examine the impact of DM on patients with gout, specifically focusing on prevalence, the associated comorbidity profile, and its effect on readmissions. We anticipated a poorer comorbidity profile in patients who have both gout and DM, particularly concerning cardiovascular and renal diseases. Additionally, a harmful effect of the combination on the risk of readmission and the development of new comorbidities is presumed.

## 2. Methods

### 2.1. Study Design, Population, and Study Variables

We designed an observational, longitudinal, population-based study. The project was approved by the Alicante-General Hospital Health Department ethics committee (ref. PI2022-113) and the Miguel Hernandez University of Elche Responsible Research Office (ref. TFG.GME.MAC.PMV.231214). As data was retrospective and pseudonymized, an exemption was granted from obtaining informed consent from the participants.

We used data from the Minimum Basic Data Set (MBDS), an administrative registry of the National Institute of Statistics in Spain. This contains information on individual care episodes, including date of birth, sex, diagnoses, medical-surgical procedures, and hospitalization outcomes, extracted from the discharge reports of public hospitals.

The study sample was extracted from the entire dataset based on specific inclusion criteria: participants had to be adults (aged 18 years and older) and have a gout diagnosis, whether as a primary or secondary condition. When coded as a primary diagnosis, it was considered the primary reason for hospital admission. Gout diagnoses were coded using the International Classification of Diseases, Ninth Edition (ICD-9), with the following codes: 274.0, 274.00, 274.01, 274.02, 274.03, 274.1, 274.10, 274.11, 274.19, 274.8, 274.81, 274.82, 274.89, or 274.9. No exclusion criteria were applied. The study covered the period from 1 January 2005 to 31 December 2015. Subsequent years were not analysed due to the transition to ICD-10.

The primary study variable was DM and its types, registered following the following ICD-9 coding: Type 1 DM (250.01, 250.03, 250. 11, 250.13, 250.21, 250.23, 250.31, 250.33, 250.41, 250.43, 250.51, 250.53, 250.61, 250.63, 250.71, 250.73, 250.81, 250.83, 250.91 or 250.93), type 2 DM (250.00, 250.02, 250.10, 250.12, 250.20, 250.22, 250.30, 250. 32, 250.40, 250.42, 250.50, 250.52, 250.60, 250.62, 250.70, 250.72, 250.80, 250.82, 250.90 or 250.92), and other types of DM (249.0 to 249.9 for secondary diabetes mellitus; 251.3 for post-surgical hypoinsulinemia–hypoinsulinemia following total or partial pancreatectomy–postpancreatectomy hyperglycemia; 962.0 for steroid-induced diabetes; 648.0 for diabetes when complicating pregnancy, childbirth, or puerperium).

DM has been reclassified into two categories: complicated and uncomplicated. The codes for complicated DM include 250.1, 250.2, 250.3, 250.4, 250.5, 250.6, 250.7, 250.8, 250.9, 249.1, 249.2, 249.3, 249.4, 249.5, 249.6, 249.7, 249.8, and 249.9. Uncomplicated DM is coded as 250.0 and 249.0. A diagnosis of complicated DM is based on ICD-9 codes indicating accompanying vascular complications.

Secondary study variables were age at admission, sex, and the following comorbidities of interest (according to ICD-9; see complete list in the Supplementary Materials): obesity, dyslipidemia, cerebrovascular disease, coronary heart disease, arrhythmia, congestive heart failure (CHF), peripheral vascular disease, venous thromboembolism (VTE), chronic kidney disease (CKD), obstructive pulmonary disease, pneumonia, sepsis, urinary tract infection, dementia, liver disease, and other concurrent rheumatic diseases. Hypertension could not be included in the analysis because it was later found to be underrepresented in the dataset (26.3%, in contrast to 62.1–82.5% prevalence in other studies [11,22]).

To evaluate the impact of coexisting gout and DM during follow-up, two composite outcome variables were constructed: Infections (including pneumonia, sepsis, and urinary tract infection) and Cardiovascular Disease (CVD: cerebrovascular disease, coronary heart disease, CHF, and peripheral vascular disease). Individual diseases, as well as CKD and VTE, were also examined as separate outcome measures.

### 2.2. Statistical Analysis

Categorical variables such as sex, DM and its subtypes, and comorbidities were presented as frequencies and percentages. Age was expressed as the mean with standard deviation (SD). However, to facilitate comparisons, we also reclassified age into sextiles (≤70 years, 71–80 years, 81–85 years, 86–90 years, 91–95 years, and >95 years). This stratification allowed for a more nuanced understanding of age-related trends in the data. Additionally, we categorized individuals as age < 40 years, a threshold used to define early-onset gout [23].

We estimated the prevalence of DM in the overall population using 95% confidence intervals (CIs). Separate analyses were conducted for type 1, type 2, and other types of DM, as well as for complicated and uncomplicated DM based on ICD-9 codes.

The chi-squared test allowed us to compare patients with and without DM on the secondary study variables (age sextiles, sex, and comorbidities). The analyses were repeated, stratifying by types of DM (type 1, type 2, and other types of DM). Subsequently, we built multiple logistic regression models that provided better differentiation between the population with and without DM in the dataset with gout, reducing the effect of potential confounders. Sextiles of age, sex, and comorbidities were included as covariates in the model.

As a post hoc sensitivity analysis, we repeated the prevalence estimation and multiple logistic regression in the gout population showing joint aspiration coded as a procedure in the discharge letter (81.91), to reinforce the diagnosis of gout.

In a later stage of our study, we identified patients with repeat admissions during the study period, defined as new registries in the dataset after the index admission (with no time restrictions). A multiple logistic regression model enabled us to identify the independent impact of DM on further readmissions, adjusted for age in sextiles, sex, and other comorbidities. No time-dependent models (such as Cox regression) were used, as we focused on the risk of readmission rather than its timing. Later, we matched DM and non-DM by age, sex, CKD, CVD, infections, and VTE in the first tercile (2005–2008), with balanced sizes to avoid overrepresenting the non-DM group. Using the chi-squared test, we then compared the frequency of comorbidities of interest between groups in the second (2009–2012) and third (2012–2015) terciles.

Statistical analyses were performed using Google Colab (Alphabet, Mountain View, CA, USA available at: https://colab.research.google.com/ accessed on 30 November 2025), which allows running Python language, using the packages matplotlib V.3.2.2, pandas V.1.3.5, scikit-learn V.1.0.2 and statsmodels V.0.12.2. We set the significance level at *p* < 0.050.

## 3. Results

### 3.1. Prevalence of DM

The selection criteria yielded a sample size of 192,062 hospitalizations with gout, with a mean age of 84.0 years (SD 12.7, IQR 76.0–93.0), being 82.6% men (n = 158,643). The distribution per sextiles of age was as follows: 30,126 (15.7%) ≤ 70 years; 36,992 (19.3%) 71–80 years; 23,950 (12.5%) 81–85 years; 33,663 (17.5%) 86–90 years; 32,409 (16.9%) 91–95 years; and 34,929 (18.2%) > 95 years. 209 (0.11%) had <40 years of age. Gout was the primary diagnosis in 10,512 (5.5%) patients.

We identified 53,242 individuals with DM (prevalence 27.7%; 95%CI 27.5–27.9%); 41,175 were male (77.3%). The prevalence of DM per sex was 26.0% in men and 36.1% in women. Table 1 compares DM and non-DM regarding sex, age sextiles, and comorbidities. In the DM population, prevalent comorbidities include dyslipidemia, CKD, coronary heart disease, CHF, and obesity. Conversely, venous thromboembolism and concurrent rheumatic diseases were more common in the non-DM population. Arrhythmia, pneumonia, and sepsis showed no significant difference between the two groups.

By type, type 2 DM was found to be much more prevalent (27.43%; 95%CI 27.23–27.63%) than type 1 (0.13%; 95%CI 0.12–0.15%) and other types of DM (0.35%; 95%CI 0.32–0.37%). Complicated DM was identified in 19.76% (95%CI 19.43–20.10%) of the diabetic population. Comparing comorbidities between types of DM and derived complications, we noted that vascular and renal disorders, infections, and obstructive pulmonary disease were more prevalent in gout plus complicated type 2 DM (Supplementary Materials, Table S1).

### 3.2. Association with Comorbidities

**Figure 1:**
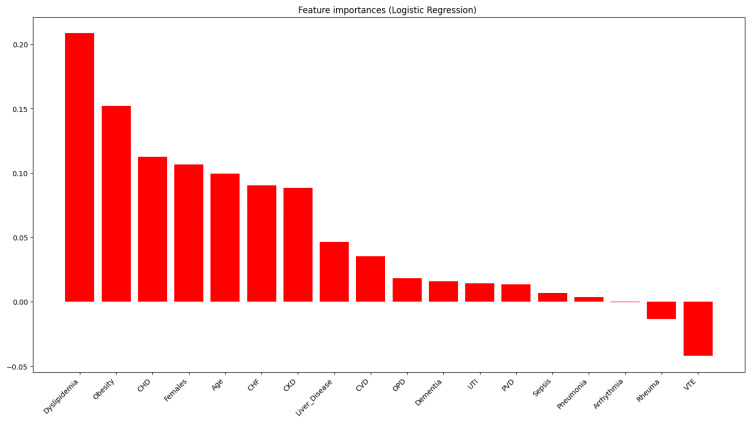
Coefficients of association between each comorbidity and diabetes mellitus, from the multiple logistic regression model. Positive coefficients indicate association with DM; negative coefficients otherwise. Legend: CHD, coronary heart disease; CHF, chronic heart failure; CKD, chronic kidney disease; CVD, cerebrovascular disease; OPD, obstructive pulmonary disease; PVD, peripheral vascular disease; UTI, urinary tract infection; VTE, venous thromboembolism.

Figure 1 and Table 2 present the results of multivariate association analyses that compare individuals with DM to those without DM in the gout dataset. The comorbidities independently associated with DM include advanced age, female sex, dyslipidemia, obesity, coronary heart disease, CHF, CKD, liver disease, cerebrovascular disease, obstructive pulmonary disease, peripheral vascular disease, urinary tract infections, and dementia. The strength of these associations varies significantly, as illustrated in Table 2. Conversely, among the non-DM population, the comorbidities identified are VTE and the presence of other concurrent rheumatic diseases.

We run separate multiple regression models for men and women (Supplementary Materials, Table S2 and Figure S1). Most of the associations persisted regardless of sex. Notably, age was not discriminative of having DM in women.

**Figure 2:**
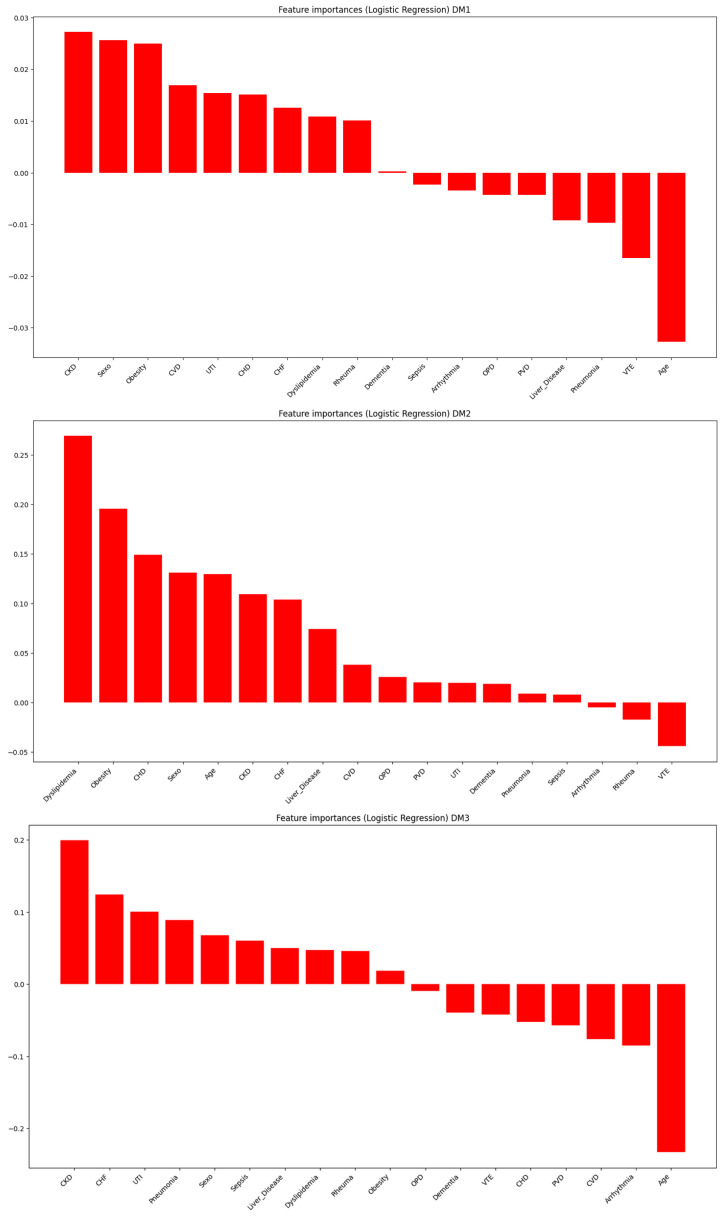
Coefficients of association between each comorbidity and diabetes mellitus, per type of diabetes (T1DM, T2DM, other DM), from the multiple logistic regression model. Positive coefficients indicate association with DM; negative coefficients otherwise. For legend, see Figure 1.

Figure 2 shows the logistic regression results stratified by type of DM. The profile of comorbidities differed significantly (Supplementary Materials, Tables S3–S5).

### 3.3. Sensitivity Analysis in the Population with Joint Aspiration Coded in the Dataset

We identified 5792 cases (85.4% men) of gout with joint aspiration as a coded procedure. In this population, the prevalence of DM was 23.4% (n = 1358), with a predominance in female patients (Supplementary Materials, Table S6). The multivariate analyses showed a pattern similar to that observed in the global population, with older age, dyslipidemia, renal disease, heart failure, and obesity being the variables most strongly associated with DM (Supplementary Materials, Figure S2).

### 3.4. Readmissions and Comorbidity Development

We identified 65,376 patients with more than one hospital admission during the study period; 16,623 (25.43%) had DM, and 48,753 (74.57%) were non-DM. Per terciles, there were 55,214 (13,513 DM, 24.47%) patients with readmission in the first tercile, 7106 patients (2129 DM, 29.96%) in the second tercile, and 3056 patients (981 DM, 32.10%) in the third tercile [*p* < 0.001]. The prevalence of complicated DM was 19.75%, 20.49%, and 22.99% in the first, second, and third terciles, respectively [*p* < 0.001]. DM was an independent predictor of subsequent admissions (adjusted odds ratio 1.04, 95%CI 1.03–1.06).

Table 3 presents the evolution of comorbidities of interest after matching the sample at the first tercile. Patients with DM had significantly more cardiovascular events over time than those without DM, mainly due to coronary heart disease and CHF. Additionally, DM was associated with a rising prevalence of CKD. Infections were similar in both groups, but VTE occurred less frequently in the diabetic population.

## 4. Discussion

In a nationwide study of adults hospitalized with gout, we found that one in four patients had DM, predominantly type 2. The prevalence was 10% higher in women than in men. Among individuals with DM, about 20% had derived complications. Multivariate analyses revealed that having DM was associated with a distinct profile of comorbidities: dyslipidemia, obesity, coronary heart disease, obstructive pulmonary disease, and heart and kidney failure were associated with DM, while VTE and concurrent rheumatic diseases were linked to the absence of DM. Type 2 DM showed a similar pattern, while other types of DM were notably linked to young ages. A longitudinal assessment highlighted that, in gout, having DM correlated with nearly a 10% higher rate of further development of cardiovascular and renal diseases compared to non-DM patients. Infection rates were comparable, but patients with DM notably had a lower risk of VTE. These findings provide valuable insights into managing patients with gout and DM, as well as help gauge the prognostic impact of their coexistence.

The prevalence of DM in the hospital-based gout population is 27.7%, nearly doubling the figures reported using global data from hospital settings [24], which underscores the strong relationship between gout and DM. Similar findings have been reported by other studies concerning the prevalence of DM in individuals with gout, primarily in outpatient settings. For instance, the French multicenter CACTUS study, which analyzed data from 2763 gout patients, found that 25% also had type 2 DM [11]. In this study, the average duration of gout was around six years. Another cross-sectional study in the UK, which included 1079 gout patients primarily from primary care, reported that 16% had DM [12]. Additionally, a case–control study by Kuo et al. showed that the prevalence of diabetes increased steadily after the diagnosis of gout [25], rising from 8.38% at baseline to 34.79% ten years later. Although gout characteristics were unavailable in the MBDS database, we assumed a long duration based on the population’s average age, as reported in a previous local study [26]. Despite being different populations, similar prevalence figures across various settings reinforce the major association between gout and DM.

Patients with gout and DM were older, more frequently women, and had particularly higher figures of dyslipidemia, obesity, and heart and kidney diseases. We had previously reported the link between female sex and DM in gout [27]. Consistent with these data, age was associated with DM only among male patients, whereas most associations persisted in the sex-stratified models. The CACTUS study found that in cluster 3, where 75% of patients had DM, they also presented dyslipidemia, hypertension, obesity, liver disease, and coronary heart disease [11]. Singh et al. also noted an association with female sex and with complications in DM, mainly hypertension, hyperlipidemia, and CKD [28]. Arterial hypertension was not analyzed due to underreporting in the dataset (already reported elsewhere for discharge letters [29]), but findings regarding other comorbidities showed a pattern similar to previous mostly outpatient gout studies. Readers should keep in mind that comorbidities, especially those managed out of the hospital, may not be well represented in a hospital discharge dataset. However, major events such as cardiovascular or renal diseases, sepsis, and other severe infections are likely well captured in the MBDS.

The inverse association between VTE and DM is noteworthy and has not been previously published. Both gout and DM pose known thrombotic risks [30,31], often linked to obesity. Recent studies have questioned the direct link between DM and VTE [32]. Reporting biases in discharge files may affect the capture of VTE cases, many of which (deep vein thromboses) are managed in outpatient clinics. Further studies are needed to thoroughly explore this issue.

Our analysis of patients with recurrent admissions revealed that concurrent DM significantly affects the development of heart and kidney diseases in a gout population. A study by Mikuls et al. involving nearly 6 million US patients with gout found that having both gout and DM increased the risk of lower limb amputation (LLA) (hazard ratio 3.36) [33], surpassing the risk associated with isolated gout (HR 1.56). Research by Singh et al. also showed that the combination of gout and DM raises the risk of myocardial infarction (HR 1.35) and stroke (HR 1.42) [28]. A Taiwanese cohort study identified DM as the most significant comorbidity in patients with gout and noted a higher incidence of stroke among women [13]. In the case–control study by Kuo et al. [25], the prevalence of complicated DM, linked to CKD and CVD, rose from 15.5% at the time of gout diagnosis to 40.0% ten years later. Similarly, the distribution of complicated DM per terciles of follow-up in our dataset slightly escalated. These findings highlight the role of DM in increasing cardiovascular and renal risk among patients with gout. Therefore, prioritizing the diagnosis and management of DM can significantly reduce the risk of related comorbidities and improve outomes in patiens with gout.

The sodium–glucose cotransporter-2 inhibitors (SGLT2i) are significant breakthrough therapies for patients with DM, CHF, or CKD [34,35]. SGLT2i improved cardiovascular mortality, renal function, and glycemic control while reducing the need for diuretics and proteinuria. Observational data indicate that they may benefit individuals with gout by decreasing gout flares, serum urate levels, cardiovascular events, and mortality compared to other anti-diabetic agents [36,37,38]. The inhibition of GLUT9b and, to a lesser extent, URAT1 transporters is responsible for increasing urine urate excretion, thereby reducing serum levels [39]. We found a significant association between DM and cardiovascular and renal comorbidities in gout patients, indicating that SGLT2i may be an ideal treatment option [36]. DM management has shifted from reducing glucose levels to preventing vascular and renal complications, with a recent focus on effective weight loss. Glucagon-like peptide-1 receptor agonists (GLP-1RAs) may also help reduce serum urate levels [40,41]; weight loss, enhanced natriuresis, and changes in insulin resistance or low-grade inflammation are potential explanations for this effect. Our study reviewed hospitalization data up to 2015, before SGLT2i and GLP-1RA were available in Spain. However, these agents may benefit patients with DM who have hyperuricemia and gout; nevertheless, formal recommendations should come from interventional studies.

Our study has several strengths. The large sample size of over 192,000 cases enabled robust multivariate models that effectively controlled for confounding factors. Additionally, the sample size permitted the assessment of previously unreported associations, including those with obstructive pulmonary disease, dementia, infectious conditions, or thromboembolic events. We also conducted a matched longitudinal analysis with 65,000 subjects with recurrent admissions, supporting causal associations. Since type 2 DM was the most prevalent in the dataset, findings for other types of DM may be less reliable due to smaller sample sizes. Similarly, the longitudinal analysis was insufficiently powered to stratify by type of DM. The dataset represents the Spanish population and uses the MBDS with ICD-9 coding, which has proven useful in previous studies [42]. However, some comorbidities that are troublesome for the patient but have a lower impact on hospitalizations (such as neuropathy or arthropathy) might be underrepresented in the dataset. Gout and comorbidities data rely on the information present in the hospital discharge reports and, therefore, on the thoroughness in completing them. Despite the large sample size likely compensating, this is a common issue in big data and should be considered when appraising our findings [43]. To ensure proper case identification, we selected all gout-relevant ICD-9 codes, despite some coding of rare presentations. We were unable to match cases with gout-related treatments (colchicine, allopurinol, febuxostat) or laboratory features (serum urate levels) because these data were not available in the dataset. However, we performed a sensitivity analysis in gout cases with documented joint aspiration (the gold-standard diagnostic procedure for gout [44]), yielding similar findings on gout prevalence and comorbidity profile, which is reassuring. Most cases included in the dataset were of advanced age, which hampers the analysis of young populations with gout. In this setting, disease pathophysiology and the occurrence of comorbidities differ and warrant further focused research. The lack of access to information on drug prescriptions and toxic substance use limits data interpretation. The study focused on data up to 2015, as the transition to ICD-10 in 2016 introduced potential mismatches. Future studies should use more recent datasets to replicate these findings. Additionally, only hospital-based data was available for mortality and comorbidities, excluding patients who died at home or did not require readmission, which may limit the applicability of the findings to community gout patients.

## 5. Conclusions

A national analysis of 11 years of hospitalizations for gout showed a unique comorbidity profile linked to DM, particularly type 2 DM, that affected one in four patients. This group, especially of advanced age and women, is more prone to cardiovascular and renal diseases. The comorbidity profile was mostly unvaried in a sex-stratified approach, but age was not a predictor of DM in women. Individuals with DM faced higher rates of readmissions and were at greater risk for vascular and renal complications, while VTE was more common in those without DM, an unreported finding to date. The coexistence of gout and DM was not observed to be more susceptible to infections. These findings highlight the need for early diagnosis and targeted management of DM in patients with gout to prevent cardiovascular–renal complications and promote weight loss.

## Figures and Tables

**Table 1:** Distribution of quintiles of age, female sex, and comorbidities of interest in populations with or without diabetes mellitus.

	DM [n = 53,242]	No DM [n = 138,820]	*p*
**Age**			**<0.001**
≤70 years	5199 (9.8)	24,927 (18.0)	
71–80 years	10,492 (19.7)	26,500 (19.1)	
81–85 years	7707 (14.5)	16,243 (11.7)	
86–90 years	10,932 (20.5)	22,728 (16.4)	
91–95 years	10,045 (18.9)	22,361 (16.1)	
>95 years	8867 (16.7)	26,061 (18.8)	
**Women**	12,067 (22.7)	21,327 (15.4)	**<0.001**
**Obesity**	9090 (17.1)	13,438 (9.7)	**<0.001**
**Dyslipidemia**	21,750 (40.9)	37,508 (27.0)	**<0.001**
**Cerebrovascular disease**	2169 (4.1)	4621 (3.3)	**<0.001**
**Coronary heart disease**	14,341 (26.9)	25,799 (18.6)	**<0.001**
**Arrhythmia**	660 (1.2)	1686 (1.2)	0.671
**Congestive heart failure**	13,034 (24.5)	24,003 (17.3)	**<0.001**
**Peripheral vascular disease**	1998 (3.8)	4367 (3.1)	**<0.001**
**Venous thromboembolism**	1000 (1.9)	3949 (2.8)	**<0.001**
**Chronic kidney disease**	16,959 (31.9)	34,148 (24.6)	**<0.001**
**Obstructive pulmonary disease**	4426 (8.3)	10,415 (7.5)	**<0.001**
**Pneumonia**	2375 (4.5)	5966 (4.3)	0.119
**Sepsis**	786 (1.5)	2094 (1.5)	0.619
**Urinary tract infection**	3752 (7.0)	8745 (6.3)	**<0.001**
**Dementia**	846 (1.6)	1766 (1.3)	**<0.001**
**Liver Disease**	1290 (2.4)	2684 (1.9)	**<0.001**
**Other rheumatic diseases**	792 (1.5)	2365 (1.7)	**<0.001**

Data is shown as n (%). DM: diabetes mellitus. In bold, statistical significance.

**Table 2:** Multivariate regression model to discriminate DM in the hospitalized population with gout.

	Coefficient	Adjusted Odds Ratio	95%CI	*p*
**Age (per year)**	+0.01	1.11	1.10–1.11	**<0.001**
**Women**	+0.35	1.11	1.11–1.12	**<0.001**
**Obesity**	+0.61	1.16	1.16–1.17	**<0.001**
**Dyslipidemia**	+0.60	1.23	1.23–1.24	**<0.001**
**Cerebrovascular diseases**	+0.27	1.04	1.03–1.04	**<0.001**
**Coronary heart disease**	+0.34	1.12	1.12–1.12	**<0.001**
**Arrhythmia**	−0.07	1.00	1.00–1.00	0.235
**Congestive heart failure**	+0.26	1.09	1.10–1.10	**<0.001**
**Peripheral vascular disease**	+0.12	1.01	1.01–1.02	**<0.001**
**Venous thromboembolism**	−0.32	0.95	0.96–0.96	**<0.001**
**Chronic kidney disease**	+0.24	1.09	1.09–1.10	**<0.001**
**Obstructive pulmonary disease**	+0.10	1.02	1.01–1.02	**<0.001**
**Pneumonia**	−0.01	1.00	1.00–1.01	0.859
**Sepsis**	+0.04	1.01	1.00–1.01	0.417
**Urinary tract infection**	+0.07	1.01	1.01–1.02	**0.005**
**Dementia**	+0.15	1.02	1.01–1.02	**0.005**
**Liver Disease**	+0.56	1.04	1.04–1.05	**<0.001**
**Other rheumatic diseases**	−0.18	0.99	0.98–0.99	**<0.001**

CI: confidence intervals. In bold, statistical significance. Odds ratios over 1.00 indicate association with DM in the study population, while odds ratios below 1.00 state otherwise.

**Table 3:** Evolution of comorbidities in gout patients with recurrent admissions over the study period, stratified by the presence of diabetes mellitus.

	Tercil 1 (2005–2008)		Tercil 2 (2009–2012)		Tercil 3 (2012–2015)		
	DM [n = 13,511]	No DM [n = 13,075]	DM [n = 1687]	No DM [n = 2280]	DM [n = 611]	No DM [n = 889]	*p*
**Cardiovascular disease (composite)**	6593 (48.8)	6169 (47.2)	934 (55.4)	1033 (45.3)	354 (57.9)	388 (43.6)	**<0.001**
**Cerebrovascular diseases**	544 (4.0)	545 (4.2)	70 (4.1)	103 (4.5)	29 (4.7)	46 (5.2)	0.530
**Coronary heart disease**	4029 (29.8)	3473 (26.6)	505 (29.9)	565 (24.8)	200 (32.7)	193 (21.7)	**<0.001**
**Congestive heart failure**	3077 (22.8)	2850 (21.8)	522 (31.0)	534 (23.4)	200 (32.7)	187 (21.0)	**<0.001**
**Peripheral vascular disease**	522 (3.9)	500 (3.8)	68 (4.0)	86 (3.8)	27 (4.4)	43 (4.8)	0.709
**Infections (composite)**	1415 (10.5)	1381 (10.6)	199 (11.8)	283 (12.4)	81 (13.3)	123 (13.8)	**<0.001**
**Pneumonia**	524 (3.9)	515 (3.9)	83 (4.9)	94 (4.1)	31 (5.1)	45 (5.1)	0.119
**Sepsis**	89 (0.7)	94 (0.7)	22 (1.3)	40 (1.8)	12 (2.0)	19 (2.1)	**<0.001**
**Urinary Tract Infection**	855 (6.3)	832 (6.4)	107 (6.3)	170 (7.5)	43 (7.0)	68 (7.6)	0.238
**Venous thromboembolism**	262 (1.9)	347 (2.7)	27 (1.6)	46 (2.0)	14 (2.3)	31 (3.5)	**<0.001**
**Chronic Kidney Disease**	2621 (19.4)	2427 (18.6)	717 (42.5)	912 (40.0)	289 (47.3)	339 (38.1)	**<0.001**

Data shown as n (%). DM: diabetes mellitus. In bold, statistical significance.

## Data Availability

Data are available upon reasonable request from the researchers.

## Supplementary Materials

The following supporting information can be downloaded at: https://www.mdpi.com/article/10.3390/jcm15051925/s1, Table S1: Distribution of quintiles of age, female sex, and comorbidities of interest per type of diabetes and presence or absence of related complications. Table S2: Multiple regression model to discriminate diabetes mellitus in the hospitalized population with gout, stratified by sex. Table S3: Multiple regression model to discriminate type 1 diabetes mellitus in the hospitalized population with gout. Table S4: Multiple regression model to discriminate type 2 diabetes mellitus in the hospitalized population with gout. Table S5: Multiple regression model to discriminate other forms of diabetes mellitus in the hospitalized population with gout. Table S6: Distribution of the prevalence of diabetes mellitus per sextiles of age and sex. Figure S1: Coefficients of association between each comorbidity and diabetes mellitus, from the multiple logistic regression model, stratified by sex. Figure S2: Coefficients of association between each comorbidity and diabetes mellitus, from the multiple logistic regression model, in the gout population restricted to having joint aspiration as a coded diagnosis.

## References

[1] Dehlin M., Jacobsson L., Roddy E. (2020). Global epidemiology of gout: Prevalence, incidence, treatment patterns and risk factors. Nat. Rev. Rheumatol..

[2] So A.K., Martinon F. (2017). Inflammation in gout: Mechanisms and therapeutic targets. Nat. Rev. Rheumatol..

[3] Dalbeth N., Gosling A.L., Gaffo A., Abhishek A. (2021). Gout. Lancet.

[4] Richette P., Doherty M., Pascual E., Barskova V., Becce F., Castañeda-Sanabria J., Coyfish M., Guillo S., Jansen T.L., Janssens H. (2017). 2016 updated EULAR evidence-based recommendations for the management of gout. Ann. Rheum. Dis..

[5] Meek I.L., Picavet H.S.J., Vonkeman H.E., Verschuren W.M.M., van de Laar M.A.F.J. (2013). Increased cardiovascular risk factors in different rheumatic diseases compared with the general population. Rheumatology.

[6] Clarson L.E., Hider S.L., Belcher J., Heneghan C., Roddy E., Mallen C.D. (2015). Increased risk of vascular disease associated with gout: A retrospective, matched cohort study in the UK clinical practice research datalink. Ann. Rheum. Dis..

[7] Stack A.G., Hanley A., Casserly L.F., Cronin C., Abdalla A., Kiernan T., Murthy B., Hegarty A., Hannigan A., Nguyen H. (2013). Independent and conjoint associations of gout and hyperuricaemia with total and cardiovascular mortality. QJM.

[8] Choi H.K., Curhan G. (2007). Independent impact of gout on mortality and risk for coronary heart disease. Circulation.

[9] Clarson L.E., Chandratre P., Hider S.L., Belcher J., Heneghan C., Roddy E., Mallen C. (2015). Increased cardiovascular mortality associated with gout: A systematic review and meta-analysis. Eur. J. Prev. Cardiol..

[10] Pérez Ruiz F., Richette P., Stack A.G., Karra Gurunath R., García de Yébenes M.J., Carmona L. (2019). Failure to reach uric acid target of <0.36 mmol/L in hyperuricaemia of gout is associated with elevated total and cardiovascular mortality. RMD Open.

[11] Richette P., Clerson P., Périssin L., Flipo R.M., Bardin T. (2015). Revisiting comorbidities in gout: A cluster analysis. Ann. Rheum. Dis..

[12] Bevis M., Blagojevic-Bucknall M., Mallen C., Hider S., Roddy E. (2018). Comorbidity clusters in people with gout: An observational cohort study with linked medical record review. Rheumatology.

[13] Huang H.C., Chiang H.P., Hsu N.W., Huang C.F., Chang S.H., Lin K.C. (2019). Differential risk group of developing stroke among older women with gouty arthritis: A latent transition analysis. Eur. J. Clin. Investig..

[14] Liu S., Sun H., Yang S., Liang N., Gao Y., Qu S., Chen H. (2024). Clustering of gout-related comorbidities and their relationship with gout flares: A data-driven cluster analysis of eight comorbidities. J. Endocrinol. Investig..

[15] Alduraibi F.K., Saleem M., Ricart K., Patel R.P., Szalai A.J., Singh J.A. (2023). Clustering Patients with Gout Based on Comorbidities and Biomarkers: A Cross-Sectional Study. J. Rheumatol..

[16] Peral-Garrido M.L., Gómez-Sabater S., Caño R., Bermúdez-García A., Boix P., Lozano T., Sánchez-Ortiga R., Perdiguero M., Caro-Martínez E., Ruiz-García C. (2025). Systemic inflammation in asymptomatic hyperuricaemia with sonographic crystal deposits, including a comparison with normouricaemia and gout. Rheumatology.

[17] Yanai H., Adachi H., Hakoshima M., Katsuyama H. (2021). Molecular Biological and Clinical Understanding of the Pathophysiology and Treatments of Hyperuricemia and Its Association with Metabolic Syndrome, Cardiovascular Diseases and Chronic Kidney Disease. Int. J. Mol. Sci..

[18] Nakagawa T., Hu H., Zharikov S., Tuttle K.R., Short R.A., Glushakova O., Ouyang X., Feig D.I., Block E.R., Herrera-Acosta J. (2006). A causal role for uric acid in fructose-induced metabolic syndrome. Am. J. Physiol. Ren. Physiol..

[19] Jamnik J., Rehman S., Blanco Mejia S., de Souza R.J., A Khan T., A Leiter L., Wolever T.M.S., Kendall C.W.C., A Jenkins D.J., Sievenpiper J.L. (2016). Fructose intake and risk of gout and hyperuricemia: A systematic review and meta-analysis of prospective cohort studies. BMJ Open.

[20] Takada T., Miyata H., Toyoda Y., Nakayama A., Ichida K., Matsuo H. (2024). Regulation of Urate Homeostasis by Membrane Transporters. Gout Urate Cryst. Depos. Dis..

[21] Choi H.K., McCormick N., Yokose C. (2022). Excess comorbidities in gout: The causal paradigm and pleiotropic approaches to care. Nat. Rev. Rheumatol..

[22] Andrés M., Bernal J.A., Sivera F., Quilis N., Carmona L., Vela P., Pascual E. (2017). Cardiovascular Risk of Patients with Gout Seen at Rheumatology Clinics Following a Structured Assessment. Ann. Rheum. Dis..

[23] Zaidi F., Narang R.K., Phipps-Green A., Gamble G.G., Tausche A.-K., So A., Riches P., Andres M., Perez-Ruiz F., Doherty M. (2020). Systematic Genetic Analysis of Early-Onset Gout: ABCG2 Is the Only Associated Locus. Rheumatology.

[24] Wallymahmed M.E., Dawes S., Clarke G., Saunders S., Younis N., MacFarlane I.A. (2005). Hospital in-patients with diabetes: Increasing prevalence and management problems. Diabet. Med..

[25] Kuo C.F., Grainge M.J., Mallen C., Zhang W., Doherty M. (2016). Comorbidities in patients with gout prior to and following diagnosis: Case-control study. Ann. Rheum. Dis..

[26] Calabuig I., Gómez-Garberí M., Andrés M. (2020). Gout Is Prevalent but Under-Registered Among Patients with Cardiovascular Events: A Field Study. Front. Med..

[27] Rodríguez-Sosa E., De Miguel E., Borrás F., Andrés M. (2023). Filling gaps in female gout: A cross-sectional study of comorbidities in 192 037 hospitalised patients. RMD Open.

[28] Singh J.A., Ramachandaran R., Yu S., Yang S., Xie F., Yun H., Zhang J., Curtis J.R. (2017). Is gout a risk equivalent to diabetes for stroke and myocardial infarction? A retrospective claims database study. Arthritis Res. Ther..

[29] Jiang J., Southern D., Beck C.A., James M., Lu M., Quan H. (2016). Validity of Canadian Discharge Abstract Data for Hypertension and Diabetes from 2002 to 2013. CMAJ Open..

[30] Cipolletta E., Tata L.J., Nakafero G., Avery A.J., Mamas M.A., Abhishek A. (2023). Risk of Venous Thromboembolism with Gout Flares. Arthritis Rheumatol..

[31] Bai J., Ding X., Du X., Zhao X., Wang Z., Ma Z. (2015). Diabetes is associated with increased risk of venous thromboembolism: A systematic review and meta-analysis. Thromb. Res..

[32] Hu S., Tan J.S., Hu M.J., Guo T.-T., Chen L., Hua L., Cao J. (2023). The Causality between Diabetes and Venous Thromboembolism: A Bidirectional Two-Sample Mendelian Randomization Study. Thromb. Haemost..

[33] Mikuls T.R., Soto Q., Petro A., Helget L., Roul P., Sayles H., Cope B., Neogi T., LaMoreaux B., O’Dell J.R. (2022). Comparison of Rates of Lower Extremity Amputation in Patients with and Without Gout in the US Department of Veterans Affairs Health System. JAMA Netw. Open.

[34] Heerspink H.J.L., Perkins B.A., Fitchett D.H., Husain M., Cherney D.Z.I. (2016). Sodium Glucose Cotransporter 2 Inhibitors in the Treatment of Diabetes Mellitus: Cardiovascular and Kidney Effects, Potential Mechanisms, and Clinical Applications. Circulation.

[35] Zelniker T.A., Braunwald E. (2020). Mechanisms of Cardiorenal Effects of Sodium-Glucose Cotransporter 2 Inhibitors: JACC State-of-the-Art Review. J. Am. Coll. Cardiol..

[36] Fralick M., Chen S.K., Patorno E., Kim S.C. (2020). Assessing the Risk for Gout with Sodium-Glucose Cotransporter-2 Inhibitors in Patients with Type 2 Diabetes: A Population-Based Cohort Study. Ann. Intern. Med..

[37] McCormick N., Yokose C., Lu N., Wexler D.J., Aviña-Zubieta J.A., De Vera M.A., McCoy R.G., Choi H.K. (2024). Sodium-Glucose Cotransporter-2 Inhibitors vs Sulfonylureas for Gout Prevention Among Patients with Type 2 Diabetes Receiving Metformin. JAMA Intern. Med..

[38] Wei J., Choi H.K., Dalbeth N., Li X., Li C., Zeng C., Lei G., Zhang Y. (2023). Gout Flares and Mortality After Sodium-Glucose Cotransporter-2 Inhibitor Treatment for Gout and Type 2 Diabetes. JAMA Netw. Open.

[39] Yokose C., McCormick N., Abhishek A., Dalbeth N., Pascart T., Lioté F., Gaffo A., FitzGerald J., Terkeltaub R., Sise M.E. (2024). The clinical benefits of sodium-glucose cotransporter type 2 inhibitors in people with gout. Nat. Rev. Rheumatol..

[40] Moreno-Pérez O., Tejera-Muñoz A., Carreño-Valdivia R., Rodríguez-Bedoya M., Guillén-Morote C., Roldán-Sánchez A., Andrés M. (2025). Impact of oral semaglutide on serum urate levels in people with type 2 diabetes: A retrospective real-world analysis (URISEMA study). Semin. Arthritis Rheum..

[41] Sattar N., Scilletta S., Stefanski A., Wang H., Daly J.W., Linetzky B. (2025). Tirzepatide and change in uric acid and its association with weight reduction: Post hoc analyses of the SURMOUNT-1 randomised placebo-controlled trial. Ann Rheum. Dis..

[42] Guzon-Illescas O., Perez Fernandez E., Crespí Villarias N., Donate F.J.Q., Peña M., Alonso-Blas C., García-Vadillo A., Mazzucchelli R. (2019). Mortality after osteoporotic hip fracture: Incidence, trends, and associated factors. J. Orthop. Surg. Res..

[43] Hohmann E. (2022). Editorial Commentary: Big Data and Machine Learning in Medicine. Arthroscopy.

[44] Richette P., Doherty M., Pascual E., Barskova V., Becce F., Castaneda J., Coyfish M., Guillo S., Jansen T., Janssens H. (2020). 2018 updated European League Against Rheumatism evidence-based recommendations for the diagnosis of gout. Ann. Rheum. Dis..

