# Supplementary material for: Prevalence and Impact of Diabetes Mellitus in Gout: Analysis of a Nationwide Dataset of 192,062 Hospitalizations
Source: J Clin Med. 2026 Mar 3;15(5):1925. doi: 10.3390/jcm15051925 (PMC12985452; doi:10.3390/jcm15051925)

Prevalence And Impact Of Diabetes Mellitus In Gout: Analysis Of A Nationwide Dataset Of 192,062 Hospitalizations

SUPPLEMENTARY MATERIAL

INDEX

Page 2. List Of International Classification Of Diseases (Icd) Ninth Edition Codes Of Comorbidities Included In The Study.

Page 26. Table S1. Distribution of quintiles of age, female sex, and comorbidities of interest per type of diabetes and presence or absence of related complications.

Page 27. Table S2. Multiple regression model to discriminate diabetes mellitus in the hospitalized population with gout, stratified by sex.

Page 28. Figure S1. Coefficients of association between each comorbidity and diabetes mellitus, from the multiple logistic regression model, stratified by sex.

Page 29. Table S3. Multiple regression model to discriminate type 1 diabetes mellitus in the hospitalized population with gout.

Page 30. Table S4. Multiple regression model to discriminate type 2 diabetes mellitus in the hospitalized population with gout.

Page 31. Table S5. Multiple regression model to discriminate other forms of diabetes mellitus in the hospitalized population with gout.

Page 32. Table S6. Distribution of the prevalence of diabetes mellitus per sextiles of age and sex.

Page 33. Figure S2. Coefficients of association between each comorbidity and diabetes mellitus, from the multiple logistic regression model, in the gout population restricted to having joint aspiration as a coded diagnosis.

LIST OF INTERNATIONAL CLASSIFICATION OF DISEASES (ICD) NINTH EDITION CODES OF COMORBIDITIES INCLUDED IN THE STUDY.

- 274 Gout:
  - 274.0 Gouty arthropathy
    - 274.00 Gouty arthropathy, unspecified
    - 274.01 Acute gouty arthropathy
    - 274.02 Chronic gouty arthropathy without mention of tophus (tophi)
    - 274.03 Chronic gouty arthropathy with tophus (tophi)
  - 274.1 Gouty nephropathy
    - 274.10 Gouty nephropathy, unspecified
    - 274.11 Uric acid nephrolithiasis
    - 274.19 Other
  - 274.8 Gout with other specified manifestations
    - 274.81 Gouty tophi of ear
    - 274.82 Gouty tophi of other sites
    - 274.89 Other
  - 274.9 Gout, unspecified
- 250 Diabetes mellitus
  - 250.0 Diabetes mellitus without mention of complication
    - 250.00 Type II or unspecified type, not stated as uncontrolled
    - 250.01 Type I [juvenile type], not stated as uncontrolled
    - 250.02 Type II or unspecified type, uncontrolled
    - 250.03 Type I [juvenile type], uncontrolled
  - 250.1 Diabetes with ketoacidosis
    - 250.10 Type II or unspecified type, not stated as uncontrolled
    - 250.11 Type I [juvenile type], not stated as uncontrolled
    - 250.12 Type II or unspecified type, uncontrolled
    - 250.13 Type I [juvenile type], uncontrolled

- 250.2 Diabetes with hyperosmolarity
  - 250.20 Type II or unspecified type, not stated as uncontrolled
  - 250.21 Type I [juvenile type], not stated as uncontrolled
  - 250.22 Type II or unspecified type, uncontrolled
  - 250.23 Type I [juvenile type], uncontrolled
- 250.3 Diabetes with other coma
  - 250.30 Type II or unspecified type, not stated as uncontrolled
  - 250.31 Type I [juvenile type], not stated as uncontrolled
  - 250.32 Type II or unspecified type, uncontrolled
  - 250.33 Type I [juvenile type], uncontrolled
- 250.4 Diabetes with renal manifestations
  - 250.40 Type II or unspecified type, not stated as uncontrolled
  - 250.41 Type I [juvenile type], not stated as uncontrolled
  - 250.42 Type II or unspecified type, uncontrolled
  - 250.43 Type I [juvenile type], uncontrolled
- 250.5 Diabetes with ophthalmic manifestations
  - 250.50 Type II or unspecified type, not stated as uncontrolled
  - 250.51 Type I [juvenile type], not stated as uncontrolled
  - 250.52 Type II or unspecified type, uncontrolled
  - 250.53 Type I [juvenile type], uncontrolled
- 250.6 Diabetes with neurological manifestations
  - 250.60 Type II or unspecified type, not stated as uncontrolled
  - 250.61 Type I [juvenile type], not stated as uncontrolled
  - 250.62 Type II or unspecified type, uncontrolled
  - 250.63 Type I [juvenile type], uncontrolled
- 250.7 Diabetes with peripheral circulatory disorders
  - 250.70 Type II or unspecified type, not stated as uncontrolled
  - 250.71 Type I [juvenile type], not stated as uncontrolled

        - 250.72 Type II or unspecified type, uncontrolled
        - 250.73 Type I [juvenile type], uncontrolled
    - 250.8 Diabetes with other specified manifestations
        - 250.80 Type II or unspecified type, not stated as uncontrolled
        - 250.81 Type I [juvenile type], not stated as uncontrolled
        - 250.82 Type II or unspecified type, uncontrolled
        - 250.83 Type I [juvenile type], uncontrolled
    - 250.9 Diabetes with unspecified complication
        - 250.90 Type II or unspecified type, not stated as uncontrolled
        - 250.91 Type I [juvenile type], not stated as uncontrolled
        - 250.92 Type II or unspecified type, uncontrolled
        - 250.93 Type I [juvenile type], uncontrolled
- 272 Dyslipidemia:
    - 272.0 Pure hypercholesterolemia
    - 272.1 Pure hyperglyceridemia
    - 272.2 Mixed hyperlipidemia
    - 272.3 Hyperchylomicronemia
    - 272.4 Other and unspecified hyperlipidemia
    - 272.9 Unspecified disorder of lipoid metabolism
- 278 Obesity:
    - 278.0 Overweight and obesity
        - 278.00 Obesity, unspecified
        - 278.01 Morbid obesity
        - 278.02 Overweight
        - 278.03 Obesity hypoventilation syndrome
    - 278.1 Localized adiposity
    - 278.8 Other hyperalimentation

- Dementia
    - 290 Dementias
        - 290.0 Senile dementia, uncomplicated
        - 290.1 Presenile dementia
            - 290.10 Presenile dementia, uncomplicated
            - 290.11 Presenile dementia with delirium
            - 290.12 Presenile dementia with delusional features
            - 290.13 Presenile dementia with depressive features
        - 290.2 Senile dementia with delusional or depressive features
            - 290.20 Senile dementia with delusional features
            - 290.21 Senile dementia with depressive features
        - 290.3 Senile dementia with delirium
        - 290.4 Vascular dementia
            - 290.40 Vascular dementia, uncomplicated
            - 290.41 Vascular dementia with delirium
            - 290.42 Vascular dementia with delusions
            - 290.43 Vascular dementia with depressed mood
        - 290.8 Other specified senile psychotic conditions
        - 290.9 Unspecified senile psychotic condition
    - 294 Persistent mental disorders due to conditions classified elsewhere
        - 294.0 Amnestic disorder in conditions classified elsewhere
        - 294.1 Dementia in conditions classified elsewhere
            - 294.10 Dementia in conditions classified elsewhere without behavioral disturbance
            - 294.11 Dementia in conditions classified elsewhere with behavioral disturbance
        - 294.2 Dementia, unspecified
            - 294.20 Dementia, unspecified, without behavioral disturbance

- 294.21 Dementia, unspecified, with behavioral disturbance

- 294.8 Other persistent mental disorders due to conditions classified elsewhere

- 294.9 Unspecified persistent mental disorders due to conditions classified elsewhere

- Hypertension:
  - 401 Essential hypertension
    - 401.0 Malignant
    - 401.1 Benign
    - 401.9 Unspecified
  - 402 Hypertensive heart disease
    - 402.0 Malignant
      - 402.00 Without heart failure
      - 402.01 With heart failure
    - 402.1 Benign
      - 402.10 Without heart failure
      - 402.11 With heart failure
    - 402.9 Unspecified
      - 402.90 Without heart failure
      - 402.91 With heart failure
  - 403 Hypertensive chronic kidney disease
    - 403.0 Malignant
      - 403.00 With chronic kidney disease stage I through stage IV, or unspecified
      - 403.01 With chronic kidney disease stage V or end stage renal disease
    - 403.1 Benign
      - 403.10 With chronic kidney disease stage I through stage IV, or unspecified
      - 403.11 With chronic kidney disease stage V or end stage renal disease
    - 403.9 Unspecified

- 403.90 With chronic kidney disease stage I through stage IV, or unspecified

- 403.91 With chronic kidney disease stage V or end stage renal disease

- 404 Hypertensive heart and chronic kidney disease

  - 404.0 Malignant

    - 404.00 Without heart failure and with chronic kidney disease stage I through stage IV, or unspecified

    - 404.01 With heart failure and with chronic kidney disease stage I through stage IV, or unspecified

    - 404.02 Without heart failure and with chronic kidney disease stage V or end stage renal disease

    - 404.03 With heart failure and chronic kidney disease stage V or end stage renal disease

  - 404.1 Benign

    - 404.10 Without heart failure and with chronic kidney disease stage I through stage IV, or unspecified

    - 404.11 With heart failure and with chronic kidney disease stage I through stage IV, or unspecified

    - 404.12 Without heart failure and with chronic kidney disease stage V or end stage renal disease

    - 404.13 With heart failure and chronic kidney disease stage V or end stage renal disease

  - 404.9 Unspecified

    - 404.90 Without heart failure and with chronic kidney disease stage I through stage IV, or unspecified

    - 404.91 With heart failure and with chronic kidney disease stage I through stage IV, or unspecified

    - 404.92 Without heart failure and with chronic kidney disease stage V or end stage renal disease

    - 404.93 With heart failure and chronic kidney disease stage V or end stage renal disease

- 405 Secondary hypertension

  - 405.0 Malignant

                - 405.01 Renovascular
                - 405.09 Other
            - 405.1 Benign
                - 405.11 Renovascular
                - 405.19 Other
            - 405.9 Unspecified
                - 405.91 Renovascular
                - 405.99 Other
- Coronary heart disease
    - 410 Acute myocardial infarction
        - 410.0 Of anterolateral wall
            - 410.00 Episode of care unspecified
            - 410.01 Initial episode of care
            - 410.02 Subsequent episode of care
        - 410.1 Of other anterior wall
            - 410.10 Episode of care unspecified
            - 410.11 Initial episode of care
            - 410.12 Subsequent episode of care
        - 410.2 Of inferolateral wall
            - 410.20 Episode of care unspecified
            - 410.21 Initial episode of care
            - 410.22 Subsequent episode of care
        - 410.3 Of inferoposterior wall
            - 410.30 Episode of care unspecified
            - 410.31 Initial episode of care
            - 410.32 Subsequent episode of care
        - 410.4 Of other inferior wall

        - 410.40 Episode of care unspecified
        - 410.41 Initial episode of care
        - 410.42 Subsequent episode of care
    - 410.5 Of other lateral wall
        - 410.50 Episode of care unspecified
        - 410.51 Initial episode of care
        - 410.52 Subsequent episode of care
    - 410.6 True posterior wall infarction
        - 410.60 Episode of care unspecified
        - 410.61 Initial episode of care
        - 410.62 Subsequent episode of care
    - 410.7 Subendocardial infarction
        - 410.70 Episode of care unspecified
        - 410.71 Initial episode of care
        - 410.72 Subsequent episode of care
    - 410.8 Of other specified sites
        - 410.80 Episode of care unspecified
        - 410.81 Initial episode of care
        - 410.82 Subsequent episode of care
    - 410.9 Unspecified site
        - 410.90 Episode of care unspecified
        - 410.91 Initial episode of care
        - 410.92 Subsequent episode of care
- 411 Other acute and subacute forms of ischemic heart disease
    - 411.0 Postmyocardial infarction syndrome
    - 411.1 Intermediate coronary syndrome
    - 411.8 Other

- 411.81 Acute coronary occlusion without myocardial infarction
- 411.89 Other

- 412 Old myocardial infarction
- 413 Angina pectoris
  - 413.0 Angina decubitus
  - 413.1 Prinzmetal angina
  - 413.9 Other and unspecified angina pectoris
- 414 Other forms of chronic ischemic heart disease
  - 414.0 Coronary atherosclerosis
    - 414.00 Of unspecified type of vessel, native or graft
    - 414.01 Of native coronary artery
    - 414.02 Of autologous biological bypass graft
    - 414.03 Of nonautologous biological bypass graft
    - 414.04 Of artery bypass graft
    - 414.05 Of unspecified type of bypass graft
    - 414.06 Of native coronary artery of transplanted heart
    - 414.07 Of bypass graft (artery) (vein) of transplanted heart
  - 414.1 Aneurysm and dissection of heart
    - 414.10 Aneurysm of heart (wall)
    - 414.11 Aneurysm of coronary vessels
    - 414.12 Dissection of coronary artery
    - 414.19 Other aneurysm of heart
  - 414.2 Chronic total occlusion of coronary artery
  - 414.3 Coronary atherosclerosis due to lipid rich plaque
  - 414.4 Coronary atherosclerosis due to calcified coronary lesion
  - 414.8 Other specified forms of chronic ischemic heart disease
  - 414.9 Chronic ischemic heart disease, unspecified

- 427 Arrhythmia:
  - 427.0 Paroxysmal supraventricular tachycardia
  - 427.1 Paroxysmal ventricular tachycardia
  - 427.2 Paroxysmal tachycardia, unspecified
  - 427.3 Atrial fibrillation and flutter
    - 427.31 Atrial fibrillation
    - 427.32 Atrial flutter
  - 427.4 Ventricular fibrillation and flutter
    - 427.41 Ventricular fibrillation
    - 427.42 Ventricular flutter
  - 427.5 Cardiac arrest
  - 427.6 Premature beats
    - 427.60 Premature beats, unspecified
    - 427.61 Supraventricular premature beats
    - 427.69 Other
  - 427.8 Other specified cardiac dysrhythmias
    - 427.81 Sinoatrial node dysfunction
    - 427.89 Other
  - 427.9 Cardiac dysrhythmia, unspecified
- 428 Heart failure
  - 428.0 Congestive heart failure, unspecified
  - 428.1 Left heart failure
  - 428.2 Systolic heart failure
    - 428.20 Unspecified
    - 428.21 Acute
    - 428.22 Chronic
    - 428.23 Acute on chronic

- 428.3 Diastolic heart failure
    - 428.30 Unspecified
    - 428.31 Acute
    - 428.32 Chronic
    - 428.33 Acute on chronic
- 428.4 Combined systolic and diastolic heart failure
    - 428.40 Unspecified
    - 428.41 Acute
    - 428.42 Chronic
    - 428.43 Acute on chronic
- 428.9 Heart failure, unspecified

- Cerebrovascular disease:
    - 430 Subarachnoid hemorrhage
    - 431 Intracerebral hemorrhage
    - 432 Other and unspecified intracranial hemorrhage
        - 432.0 Nontraumatic extradural hemorrhage
        - 432.1 Subdural hemorrhage
        - 432.9 Unspecified intracranial hemorrhage
    - 433 Occlusion and stenosis of precerebral arteries
        - 433.0 Basilar artery
            - 433.00 Without mention of cerebral infarction
            - 433.01 With cerebral infarction
        - 433.1 Carotid artery
            - 433.10 Without mention of cerebral infarction
            - 433.11 With cerebral infarction
        - 433.2 Vertebral artery
            - 433.20 Without mention of cerebral infarction

            - 433.21 With cerebral infarction
        - 433.3 Multiple and bilateral
            - 433.30 Without mention of cerebral infarction
            - 433.31 With cerebral infarction
        - 433.8 Other specified precerebral artery
            - 433.80 Without mention of cerebral infarction
            - 433.81 With cerebral infarction
        - 433.9 Unspecified precerebral artery
            - 433.90 Without mention of cerebral infarction
            - 433.91 With cerebral infarction
    - 434 Occlusion of cerebral arteries
        - 434.0 Cerebral thrombosis
            - 434.00 Without mention of cerebral infarction
            - 434.01 With cerebral infarction
        - 434.1 Cerebral embolism
            - 434.10 Without mention of cerebral infarction
            - 434.11 With cerebral infarction
        - 434.9 Cerebral artery occlusion, unspecified
            - 434.90 Without mention of cerebral infarction
            - 434.91 With cerebral infarction
    - 435 Transient cerebral ischemia
        - 435.0 Basilar artery syndrome
        - 435.1 Vertebral artery syndrome
        - 435.2 Subclavian steal syndrome
        - 435.3 Vertebrobasilar artery syndrome
        - 435.8 Other specified transient cerebral ischemias
        - 435.9 Unspecified transient cerebral ischemia

- 436 Acute, but ill-defined, cerebrovascular disease
- 437 Other and ill-defined cerebrovascular disease
  - 437.0 Cerebral atherosclerosis
  - 437.1 Other generalized ischemic cerebrovascular disease
  - 437.2 Hypertensive encephalopathy
  - 437.3 Cerebral aneurysm, nonruptured
  - 437.4 Cerebral arteritis
  - 437.5 Moyamoya disease
  - 437.6 Nonpyogenic thrombosis of intracranial venous sinus
  - 437.7 Transient global amnesia
  - 437.8 Other
  - 437.9 Unspecified
- 438 Late effects of cerebrovascular disease
  - 438.0 Cognitive deficits
  - 438.1 Speech and language deficits
    - 438.10 Speech and language deficit, unspecified
    - 438.11 Aphasia
    - 438.12 Dysphasia
    - 438.13 Dysarthria
    - 438.14 Fluency disorder
    - 438.19 Other speech and language deficits
  - 438.2 Hemiplegia/hemiparesis
    - 438.20 Hemiplegia affecting unspecified side
    - 438.21 Hemiplegia affecting dominant side
    - 438.22 Hemiplegia affecting nondominant side
  - 438.3 Monoplegia of upper limb
    - 438.30 Monoplegia of upper limb affecting unspecified side

            - 438.31 Monoplegia of upper limb affecting dominant side
            - 438.32 Monoplegia of upper limb affecting nondominant side
        - 438.4 Monoplegia of lower limb
            - 438.40 Monoplegia of lower limb affecting unspecified side
            - 438.41 Monoplegia of lower limb affecting dominant side
            - 438.42 Monoplegia of lower limb affecting nondominant side
        - 438.5 Other paralytic syndrome
            - 438.50 Other paralytic syndrome affecting unspecified side
            - 438.51 Other paralytic syndrome affecting dominant side
            - 438.52 Other paralytic syndrome affecting nondominant side
            - 438.53 Other paralytic syndrome, bilateral
        - 438.6 Alterations of sensations
        - 438.7 Disturbances of vision
        - 438.8 Other late effects of cerebrovascular disease
            - 438.81 Apraxia
            - 438.82 Dysphagia
            - 438.83 Facial weakness
            - 438.84 Ataxia
            - 438.85 Vertigo
            - 438.89 Other late effects of cerebrovascular disease
        - 438.9 Unspecified late effects of cerebrovascular disease
- 443 Peripheral vascular disease
    - 443.0 Raynaud's syndrome
    - 443.1 Thromboangiitis obliterans [Buerger's disease]
    - 443.2 Other arterial dissection
        - 443.21 Dissection of carotid artery
        - 443.22 Dissection of iliac artery

        - 443.23 Dissection of renal artery
        - 443.24 Dissection of vertebral artery
        - 443.29 Dissection of other artery
    - 443.8 Other specified peripheral vascular diseases
        - 443.81 Peripheral angiopathy in diseases classified elsewhere
        - 443.82 Erythromelalgia
        - 443.89 Other
    - 443.9 Peripheral vascular disease, unspecified
- Venous thromboembolism:
    - 415 Acute pulmonary heart disease
        - 415.1 Pulmonary embolism and infarction
            - 415.11 Iatrogenic pulmonary embolism and infarction
            - 415.13 Saddle embolus of pulmonary artery
            - 415.19 Other
    - 416 Chronic pulmonary heart disease
        - 416.2 Chronic pulmonary embolism
    - 451 Phlebitis and thrombophlebitis
        - 451.0 Of superficial vessels of lower extremities
        - 451.1 Of deep vessels of lower extremities
            - 451.11 Femoral vein (deep) (superficial)
            - 451.19 Other
        - 451.2 Of lower extremities, unspecified
        - 451.8 Of other sites
            - 451.81 Iliac vein
            - 451.82 Of superficial veins of upper extremities
            - 451.83 Of deep veins of upper extremities
            - 451.84 Of upper extremities, unspecified

        - 451.89 Other

    - 451.9 Of unspecified site

- 452 Portal vein thrombosis

- 453 Other venous embolism and thrombosis

    - 453.0 Budd-Chiari syndrome

    - 453.1 Thrombophlebitis migrans

    - 453.2 Of inferior vena cava

    - 453.3 Of renal vein

    - 453.4 Acute venous embolism and thrombosis of deep vessels of lower extremity

        - 453.40 Acute venous embolism and thrombosis of unspecified deep vessels of lower extremity

        - 453.41 Acute venous embolism and thrombosis of deep vessels of proximal lower extremity

        - 453.42 Acute venous embolism and thrombosis of deep vessels of distal lower extremity

    - 453.5 Chronic venous embolism and thrombosis of deep vessels of lower extremity

        - 453.50 Chronic venous embolism and thrombosis of unspecified deep vessels of lower extremity

        - 453.51 Chronic venous embolism and thrombosis of deep vessels of proximal lower extremity

        - 453.52 Chronic venous embolism and thrombosis of deep vessels of distal lower extremity

    - 453.6 Venous embolism and thrombosis of superficial vessels of lower extremity

    - 453.7 Chronic venous embolism and thrombosis of other specified vessels

        - 453.71 Chronic venous embolism and thrombosis of superficial veins of upper extremity

        - 453.72 Chronic venous embolism and thrombosis of deep veins of upper extremity

        - 453.73 Chronic venous embolism and thrombosis of upper extremity, unspecified

                - 453.74 Chronic venous embolism and thrombosis of axillary veins
                - 453.75 Chronic venous embolism and thrombosis of subclavian veins
                - 453.76 Chronic venous embolism and thrombosis of internal jugular veins
                - 453.77 Chronic venous embolism and thrombosis of other thoracic veins
                - 453.79 Chronic venous embolism and thrombosis of other specified veins
            - 453.8 Acute venous embolism and thrombosis of other specified veins
                - 453.81 Acute venous embolism and thrombosis of superficial veins of upper extremity
                - 453.82 Acute venous embolism and thrombosis of deep veins of upper extremity
                - 453.83 Acute venous embolism and thrombosis of upper extremity, unspecified
                - 453.84 Acute venous embolism and thrombosis of axillary veins
                - 453.85 Acute venous embolism and thrombosis of subclavian veins
                - 453.86 Acute venous embolism and thrombosis of internal jugular veins
                - 453.87 Acute venous embolism and thrombosis of other thoracic veins
                - 453.89 Acute venous embolism and thrombosis of other specified veins
            - 453.9 Of unspecified site
        - 459 Other disorders of circulatory system
            - 459.1 Postphlebitic syndrome
                - 459.10 Postphlebitic syndrome without complications
                - 459.11 Postphlebitic syndrome with ulcer
                - 459.12 Postphlebitic syndrome with inflammation
                - 459.13 Postphlebitic syndrome with ulcer and inflammation
                - 459.19 Postphlebitic syndrome with other complication
- Pneumonia:
    - 480 Viral pneumonia

- 480.0 Pneumonia due to adenovirus
- 480.1 Pneumonia due to respiratory syncytial virus
- 480.2 Pneumonia due to parainfluenza virus
- 480.3 Pneumonia due to SARS-associated coronavirus
- 480.8 Pneumonia due to other virus not elsewhere classified
- 480.9 Viral pneumonia, unspecified

- 481 Pneumococcal pneumonia
- 482 Other bacterial pneumonia
    - 482.0 Pneumonia due to Klebsiella pneumoniae
    - 482.1 Pneumonia due to Pseudomonas
    - 482.2 Pneumonia due to Hemophilus influenzae [H. influenzae]
    - 482.3 Pneumonia due to Streptococcus
        - 482.30 Streptococcus, unspecified
        - 482.31 Group A
        - 482.32 Group B
        - 482.39 Other Streptococcus
    - 482.4 Pneumonia due to Staphylococcus
        - 482.40 Pneumonia due to Staphylococcus, unspecified
        - 482.41 Methicillin susceptible pneumonia due to Staphylococcus aureus
        - 482.42 Methicillin resistant pneumonia due to Staphylococcus aureus
        - 482.49 Other Staphylococcus pneumonia
    - 482.8 Pneumonia due to other specified bacteria
        - 482.81 Anaerobes
        - 482.82 Escherichia coli [E. coli]
        - 482.83 Other gram-negative bacteria
        - 482.84 Legionnaires' disease
        - 482.89 Other specified bacteria

        - 482.9 Bacterial pneumonia unspecified
    - 483 Pneumonia due to other specified organism
        - 483.0 Mycoplasma pneumoniae
        - 483.1 Chlamydia
        - 483.8 Other specified organism
    - 484 Pneumonia in infectious diseases classified elsewhere
        - 484.1 Pneumonia in cytomegalic inclusion disease
        - 484.3 Pneumonia in whooping cough
        - 484.5 Pneumonia in anthrax
        - 484.6 Pneumonia in aspergillosis
        - 484.7 Pneumonia in other systemic mycoses
        - 484.8 Pneumonia in other infectious diseases classified elsewhere
    - 485 Bronchopneumonia, organism unspecified
    - 486 Pneumonia, organism unspecified
- Obstructive pulmonary disease:
    - 490 Bronchitis, not specified as acute or chronic
    - 491 Chronic bronchitis
        - 491.0 Simple chronic bronchitis
        - 491.1 Mucopurulent chronic bronchitis
        - 491.2 Obstructive chronic bronchitis
        - 491.20 Without exacerbation
            - 491.21 With (acute) exacerbation
            - 491.22 With acute bronchitis
        - 491.8 Other chronic bronchitis
        - 491.9 Unspecified chronic bronchitis
    - 492 Emphysema
        - 492.0 Emphysematous bleb

        - 492.8 Other emphysema
    - 493 Asthma
        - 493.0 Extrinsic asthma
            - 493.00 Unspecified
            - 493.01 With status asthmaticus
            - 493.02 With (acute) exacerbation
        - 493.1 Intrinsic asthma
            - 493.10 Unspecified
            - 493.11 With status asthmaticus
            - 493.12 With (acute) exacerbation
        - 493.2 Chronic obstructive asthma
            - 493.20 Unspecified
            - 493.21 With status asthmaticus
            - 493.22 With (acute) exacerbation
    - 494 Bronchiectasis
        - 494.0 Bronchiectasis without acute exacerbation
        - 494.1 Bronchiectasis with acute exacerbation
    - 495 Extrinsic allergic alveolitis
        - 495.0 Farmers' lung
        - 495.1 Bagassosis
        - 495.2 Bird-fanciers' lung
        - 495.3 Suberosis
        - 495.4 Malt workers' lung
        - 495.5 Mushroom workers' lung
        - 495.6 Maple bark-strippers' lung
        - 495.7 "Ventilation" pneumonitis
        - 495.8 Other specified allergic alveolitis and pneumonitis

        - 495.9 Unspecified allergic alveolitis and pneumonitis
    - 496 Chronic airway obstruction, not elsewhere classified
- Liver disease:
    - 571 Chronic liver disease and cirrhosis
        - 571.0 Alcoholic fatty liver
        - 571.1 Acute alcoholic hepatitis
        - 571.2 Alcoholic cirrhosis of liver
        - 571.3 Alcoholic liver damage, unspecified
        - 571.4 Chronic hepatitis
            - 571.40 Chronic hepatitis, unspecified
            - 571.41 Chronic persistent hepatitis
            - 571.42 Autoimmune hepatitis
            - 571.49 Other
        - 571.5 Cirrhosis of liver without mention of alcohol
        - 571.6 Biliary cirrhosis
        - 571.8 Other chronic nonalcoholic liver disease
        - 571.9 Unspecified chronic liver disease without mention of alcohol
    - 572 Liver abscess and sequelae of chronic liver disease
        - 572.2 Hepatic encephalopathy
        - 572.3 Portal hypertension
        - 572.4 Hepatorenal syndrome
        - 572.8 Other sequelae of chronic liver disease
    - 573 Other disorders of liver
        - 573.0 Chronic passive congestion of liver
        - 573.5 Hepatopulmonary syndrome
        - 573.8 Other specified disorders of liver
        - 573.9 Unspecified disorder of liver

- 585 Chronic kidney disease
    - 585.1 Chronic kidney disease, Stage I
    - 585.2 Chronic kidney disease, Stage II (mild)
    - 585.3 Chronic kidney disease, Stage III (moderate)
    - 585.4 Chronic kidney disease, Stage IV (severe)
    - 585.5 Chronic kidney disease, Stage V
    - 585.6 End stage renal disease
    - 585.9 Chronic kidney disease, unspecified
- Urinary tract infection:
    - 590 Infections of kidney
        - 590.0 Chronic pyelonephritis
            - 590.00 Without lesion of renal medullary necrosis
            - 590.01 With lesion of renal medullary necrosis
        - 590.1 Acute pyelonephritis
            - 590.10 Without lesion of renal medullary necrosis
            - 590.11 With lesion of renal medullary necrosis
        - 590.2 Renal and perinephric abscess
        - 590.3 Pyeloureteritis cystica
        - 590.8 Other pyelonephritis or pyonephrosis, not specified as acute or chronic
            - 590.80 Pyelonephritis, unspecified
            - 590.81 Pyelitis or pyelonephritis in diseases classified elsewhere
        - 590.9 Infection of kidney, unspecified
    - 595 Cystitis
        - 595.0 Acute cystitis
        - 595.1 Chronic interstitial cystitis
        - 595.2 Other chronic cystitis
        - 595.3 Trigonitis

        - 595.4 Cystitis in diseases classified elsewhere
        - 595.8 Other specified types of cystitis
            - 595.81 Cystitis cystica
            - 595.89 Other
        - 595.9 Cystitis, unspecified
    - 597 Urethritis, not sexually transmitted, and urethral syndrome
        - 597.0 Urethral abscess
        - 597.8 Other urethritis
        - 597.80 Urethritis, unspecified
            - 597.81 Urethral syndrome NOS
            - 597.89 Other
    - 599 Other disorders of urethra and urinary tract
        - 599.0 Urinary tract infection, site not specified
- Urinary lithiasis
    - 591 Hydronephrosis
    - 592 Calculus of kidney and ureter
        - 592.0 Calculus of kidney
        - 592.1 Calculus of ureter
        - 592.9 Urinary calculus, unspecified
    - 594 Calculus of lower urinary tract
        - 594.0 Calculus in diverticulum of bladder
        - 594.1 Other calculus in bladder
        - 594.2 Calculus in urethra
        - 594.8 Other lower urinary tract calculus
        - 594.9 Calculus of lower urinary tract, unspecified
- Rheumatological disease
    - 710 Diffuse diseases of connective tissue

            - 710.0 Systemic lupus erythematosus
            - 710.1 Systemic sclerosis
            - 710.2 Sicca syndrome
            - 710.3 Dermatomyositis
            - 710.4 Polymyositis
            - 710.5 Eosinophilia myalgia syndrome
            - 710.8 Other specified diffuse diseases of connective tissue
            - 710.9 Unspecified diffuse connective tissue disease
        - 714 Rheumatoid arthritis and other inflammatory polyarthropathies
            - 714.0 Rheumatoid arthritis
            - 714.1 Felty's syndrome
            - 714.2 Other rheumatoid arthritis with visceral or systemic involvement
            - 714.3 Juvenile chronic polyarthritis
                - 714.30 Polyarticular juvenile rheumatoid arthritis, chronic or unspecified
                - 714.31 Polyarticular juvenile rheumatoid arthritis, acute
                - 714.32 Pauciarticular juvenile rheumatoid arthritis
                - 714.33 Monoarticular juvenile rheumatoid arthritis
            - 714.4 Chronic postrheumatic arthropathy
            - 714.8 Other specified inflammatory polyarthropathies
                - 714.81 Rheumatoid lung
                - 714.89 Other
            - 714.9 Unspecified inflammatory polyarthropathy
- 995 Sepsis
    - 995.91 Sepsis
    - 995.92 Severe sepsis

Table S1. *Distribution of quintiles of age, female sex, and comorbidities of interest per type of diabetes and presence or absence of related complications***.**

	**Type 1 DM** [n=253]			**Type 2 DM** [n=52689]			**Other types of DM** [n=665]		
	Complicated [N=95]	Not complicated [N=158]	*P*	Complicated [N=10288]	Not complicated [N=42401]	*P*	Complicated [N=142]	Not complicated [N=523]	*P*
**Age**			0.277			0.270			**0.015**
<=70 years	28 (29.5)	35 (22.2)		1006 (9.8)	4087 (9.6)		35 (25.0)	91 (17.4)	
71-80 years	17 (17.9)	22 (13.9)		1998 (19.4)	8377 (19.8)		31 (22.1)	139 (26.6)	
81-85 years	10 (10.5)	23 (14.6)		1468 (14.3)	6155 (14.5)		11 (7.9)	74 (14.1)	
86-90 years	9 (9.5)	30 (19.0)		2199 (21.4)	8637 (20.4)		18 (12.9)	65 (12.4)	
91-95 years	15 (15.8)	25 (15.8)		1942 (18.9)	8022 (18.9)		18 (12.9)	93 (17.8)	
>95 years	16 (16.8)	23 (14.6)		1675 (16.3)	7123 (16.8)		27 (19.3)	61 (11.7)	
**Women**	27 (28.4)	38 (24.1)	0.534	2497 (24.3)	9426 (22.2)	**0.024**	36 (25.7)	93 (17.8)	0.047
**Obesity**	21 (22.1)	32 (20.3)	0.849	1848 (18.0)	7135 (16.8)	**0.006**	19 (13.6)	64 (12.2)	0.779
**Dyslipidemia**	26 (27.4)	63 (39.9)	0.060	3801 (36.9)	17735 (41.8)	**<0.001**	44 (31.4)	171 (32.7)	0.855
**Cerebrovascular diseases**	6 (6.3)	11 (7.0)	1.000	460 (4.5)	1688 (4.0)	**0.026**	2 (1.4)	12 (2.3)	0.763
**Coronary heart disease**	22 (23.2)	40 (25.3)	0.814	2706 (26.3)	11487 (27.1)	0.108	28 (20.0)	81 (15.5)	0.250
**Arrhythmia**	3 (3.2)	0 (0.0)	0.099	104 (1.0)	549 (1.3)	**0.022**	0 (0.0)	6 (1.1)	0.441
**Congestive heart failure**	19 (20.0)	31 (19.6)	1.000	2788 (27.1)	10128 (23.9)	**<0.001**	26 (18.6)	114 (21.8)	0.475
**Peripheral vascular disease**	7 (7.4)	4 (2.5)	0.131	463 (4.5)	1510 (3.6)	**0.019**	4 (2.9)	14 (2.7)	1.000
**Venous thromboembolism**	1 (1.1)	1 (0.6)	1.000	181 (1.8)	817 (1.9)	0.281	1 (0.7)	17 (3.3)	0.178
**Chronic kidney disease**	32 (33.7)	46 (29.1)	0.534	4825 (46.9)	11907 (28.1)	**<0.001**	58 (41.4)	195 (37.3)	0.425
**Obstructive pulmonary disease**	5 (5.3)	12 (7.6)	0.647	756 (7.3)	3637 (8.6)	**0.038**	13 (9.3)	32 (6.1)	0.257
**Pneumonía**	2 (2.1)	6 (3.8)	0.708	546 (5.3)	1807 (4.3)	**0.012**	12 (8.6)	41 (7.8)	0.914
**Sepsis**	0 (0)	3 (1.9)	0.452	191 (1.9)	586 (1.4)	**<0.001**	1 (0.7)	19 (3.6)	0.130
**Urinary tract infection**	7 (7.4)	13 (8.2)	0.996	964 (9.4)	2747 (6.5)	**<0.001**	11 (7.9)	47 (9.0)	0.801
**Dementia**	2 (2.1)	4 (2.5)	1.000	149 (1.4)	687 (1.6)	0.227	4 (2.9)	4 (0.8)	0.115
**Liver Disease**	0 (0.0)	4 (2.5)	0.297	191 (1.9)	1089 (2.6)	**0.020**	1 (0.7)	15 (2.9)	0.244
**Other rheumatic diseases**	3 (3.2)	1 (0.6)	0.299	163 (1.6)	621 (1.5)	0.393	2 (1.4)	23 (4.4)	0.165

Data is shown as n (%). DM: diabetes mellitus. In bold, statistical significance.

Table S2. *Multivariate regression model to discriminate DM in the hospitalized population with gout, stratified per sex.*

	Men				***Women***			
	**Coefficient**	**aOR**	**95%CI**	***P***	**Coefficient**	**aOR**	**95%CI**	***P***
**Age (per year)**	+0.104	1.11	1.10-1.11	**<0.001**	-0.001	0.99	0.99-1.00	0.894
**Obesity**	+0.130	1.14	1.13-1.14	**<0.001**	+0.239	1.27	1.26-1.28	**<0.001**
**Dyslipidemia**	+0.192	1.21	1.20-1.21	**<0.001**	+0.249	1.28	1.27-1.29	**<0.001**
**Cerebrovascular diseases**	+0.031	1.03	1.02-1.03	**<0.001**	+0.043	1.04	1.03-1.05	**<0.001**
**Coronary heart disease**	+0.108	1.11	1.10-1.11	**<0.001**	+0.157	1.17	1.16-1.18	**<0.001**
**Arrhythmia**	+0.002	1.00	0.99-1.00	0.500	-0.022	0.97	0.97-0.98	**<0.001**
**Congestive heart failure**	+0.076	1.08	1.07-1.08	**<0.001**	+0.120	1.13	1.11-1.13	**<0.001**
**Peripheral vascular disease**	+0.020	1.02	1.01-1.02	**<0.001**	+0.011	1.01	1.00-1.01	**0.009**
**Venous thromboembolism**	-0.037	0.96	0.95-0.96	**<0.001**	-0.060	0.94	0.93-0.95	**<0.001**
**Chronic kidney disease**	+0.092	1.09	1.09-1.10	**<0.001**	+0.052	1.05	1.04-1.06	**<0.001**
**Obstructive pulmonary disease**	+0.022	1.02	1.01-1.02	**<0.001**	+0.013	1.01	1.00-1.02	**0.002**
**Pneumonia**	+0.002	1.00	0.99-1.01	0.292	+0.012	1.01	1.00-1.02	**0.003**
**Sepsis**	+0.001	1.00	0.99-1.00	0.725	+0.002	1.00	0.99-1.00	0.594
**Urinary tract infection**	+0.014	1.01	1.01-1.02	**<0.001**	+0.029	1.03	1.02-1.03	**<0.001**
**Dementia**	+0.017	1.02	1.01-1.02	**<0.001**	+0.007	1.00	0.99-1.01	0.082
**Liver Disease**	+0.043	1.04	1.03-1.04	**<0.001**	+0.101	1.10	1.09-1.11	**<0.001**
**Other rheumatic diseases**	-0.008	0.99	0.98-0.99	**<0.001**	-0.050	0.95	0.94-0.95	**<0.001**

CI: confidence intervals. aOR: adjusted odds ratio. In bold, statistical significance.
Odds ratios over 1.00 indicate association with DM in the study population, while odds ratios below 1.00 state otherwise.

Figure S1*. Coefficients of association between each comorbidity and diabetes mellitus, from the multiple logistic regression model, stratified by sex. The top image shows data restricted to male patients. The bottom image shows data restricted to female patients. Positive coefficients indicate an association with DM; negative coefficients indicate the opposite. Legend: CHD, coronary heart disease; CHF, chronic heart failure; CKD, chronic kidney disease; CVD, cerebrovascular disease; OPD, obstructive pulmonary disease; PVD, peripheral vascular disease; UTI, urinary tract infection; VTE, venous thromboembolism.*

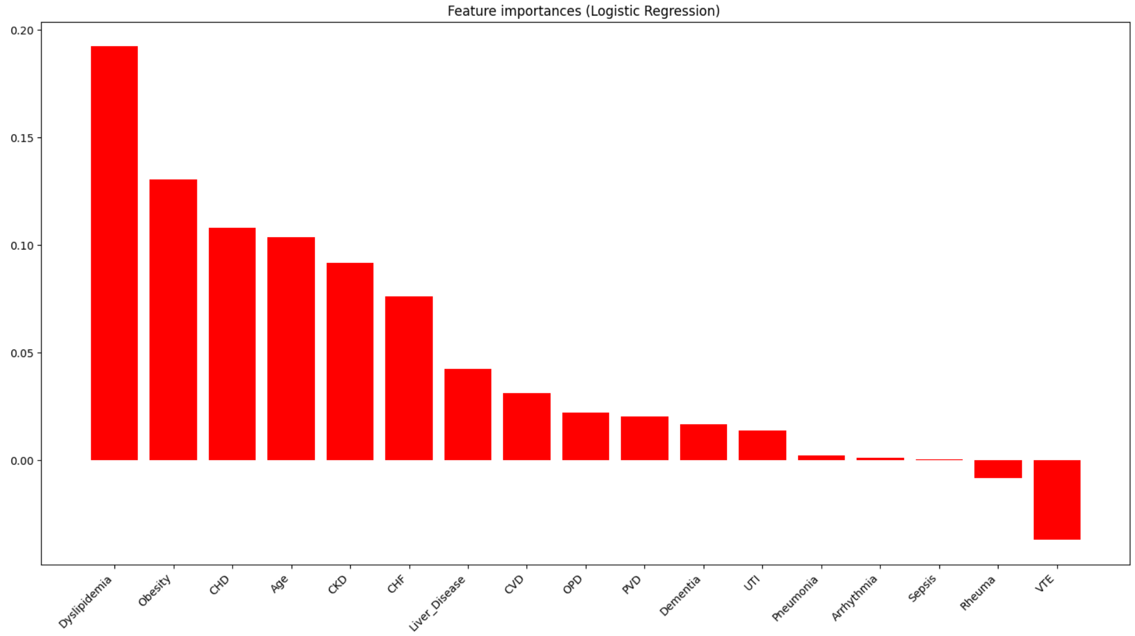

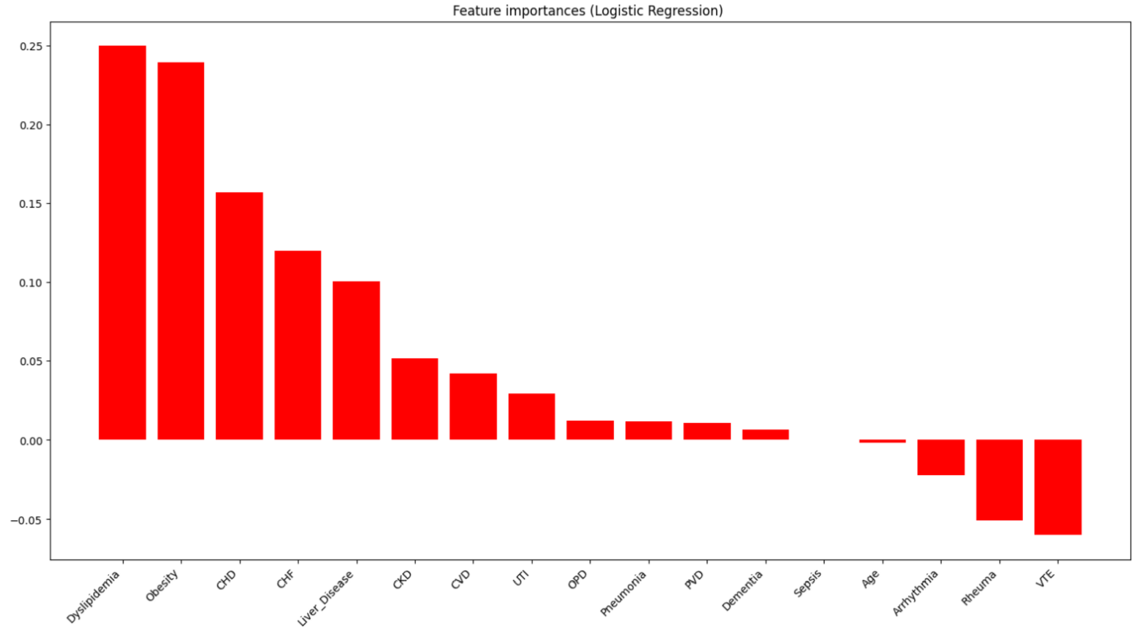

Table S3. *Multivariate regression model to discriminate type 1 diabetes mellitus in the hospitalized population with gout.*

	**Coefficient**	**Odds ratio**	**95%CI**	***P***
**Age (per year)**	-0.03	0.97	0.92-1.02	0.220
**Women**	+0.03	1.03	0.98-1.08	0.326
**Obesity**	+0.03	1.03	0.98-1.08	0.323
**Dyslipidemia**	+0.01	1.01	0.96-1.06	0.667
**Cerebrovascular disease**	+0.02	1.02	0.97-1.07	0.500
**Coronary heart disease**	+0.02	1.02	0.97-1.07	0.556
**Arrhythmia**	-0.01	1.00	0.94-1.05	0.889
**Congestive heart failure**	+0.01	1.01	0.96-1.07	0.637
**Peripheral vascular disease**	-0.01	1.00	0.95-1.05	0.866
**Venous thromboembolism**	-0.02	0.98	0.94-1.03	0.509
**Chronic kidney disease**	+0.03	1.03	0.98-1.08	0.285
**Obstructive pulmonary disease**	-0.01	1.00	0.95-1.05	0.865
**Pneumonia**	-0.01	0.99	0.94-1.04	0.689
**Sepsis**	-0.01	1.00	0.95-1.05	0.926
**Urinary tract infection**	+0.02	1.02	0.97-1.07	0.545
**Dementia**	+0.00	1.00	0.95-1.05	0.993
**Liver Disease**	-0.01	0.99	0.94-1.04	0.714
**Other rheumatic diseases**	+0.01	1.01	0.96-1.06	0.686

CI: confidence intervals. In bold, statistical significance.

Table S4. *Multivariate regression model to discriminate type 2 diabetes mellitus in the hospitalized population with gout.*

	**Coefficient**	**Odds ratio**	**95%CI**	***P***
**Age (per year)**	+0.13	1.14	1.14-1.14	**<0.001**
**Women**	+0.13	1.14	1.14-1.14	**<0.001**
**Obesity**	+0.20	1.22	1.21-1.22	**<0.001**
**Dyslipidemia**	+0.27	1.31	1.30-1.31	**<0.001**
**Cerebrovascular diseases**	+0.04	1.04	1.03-1.04	**<0.001**
**Coronary heart disease**	+0.15	1.16	1.16-1.17	**<0.001**
**Arrhythmia**	-0.01	0.99	0.99-0.99	**0.016**
**Congestive heart failure**	+0.10	1.11	1.11-1.11	**<0.001**
**Peripheral vascular disease**	+0.02	1.02	1.02-1.02	**<0.001**
**Venous thromboembolism**	-0.04	0.96	0.95-0.96	**<0.001**
**Chronic kidney disease**	+0.11	1.12	1.11-1.12	**<0.001**
**Obstructive pulmonary disease**	+0.03	1.03	1.02-1.03	**<0.001**
**Pneumonia**	+0.01	1.01	1.01-1.01	**<0.001**
**Sepsis**	+0.01	1.01	1.00-1.01	**<0.001**
**Urinary tract infection**	+0.02	1.02	1.01-1.02	**<0.001**
**Dementia**	+0.02	1.02	1.02-1.02	**<0.001**
**Liver Disease**	+0.07	1.08	1.07-1.08	**<0.001**
**Other rheumatic diseases**	-0.17	0.98	0.98-0.99	**<0.001**

CI: confidence intervals. In bold, statistical significance.

Table S5. *Multivariate regression model to discriminate other forms of diabetes mellitus in the hospitalized population with gout.*

	Coefficient	Odds ratio	95%CI	*P*
**Age (per year)**	-0.23	0.79	0.76-0.82	**<0.001**
**Women**	+0.07	1.07	1.04-1.11	**<0.001**
**Obesity**	+0.02	1.02	0.99-1.05	0.259
**Dyslipidemia**	+0.05	1.05	1.01-1.08	**0.005**
**Cerebrovascular diseases**	-0.08	0.93	0.90-0.96	**<0.001**
**Coronary heart disease**	-0.05	0.95	0.92-0.98	**0.002**
**Arrhythmia**	-0.09	0.92	0.89-0.95	**0.016**
**Congestive heart failure**	+0.12	1.13	1.09-1.17	**<0.001**
**Peripheral vascular disease**	-0.06	0.94	0.91-0.98	**0.001**
**Venous thromboembolism**	-0.04	0.96	0.93-0.99	**0.011**
**Chronic kidney disease**	+0.20	1.22	1.18-1.26	**<0.001**
**Obstructive pulmonary disease**	-0.01	0.99	0.96-1.02	0.584
**Pneumonia**	+0.09	1.09	1.06-1.13	**<0.001**
**Sepsis**	+0.06	1.06	1.03-1.10	**<0.001**
**Urinary tract infection**	+0.10	1.11	1.07-1.14	**<0.001**
**Dementia**	-0.04	0.96	0.93-0.99	**0.019**
**Liver Disease**	+0.05	1.05	1.02-1.09	**0.003**
**Other rheumatic diseases**	+0.05	1.05	1.01-1.08	**0.005**

CI: confidence intervals. In bold, statistical significance.

Table S6. *Distribution of the prevalence of diabetes mellitus per sextiles of age and sex in the gout population restricted to individuals with coded joint aspiration in the dataset.*

Age group	**Males**	**Females**
≤70 years	10.2	20.3
71-80 years	25.7	36.1
81-85 years	31.4	32.4
86-90 years	32.4	42.3
91-95 years	27.3	35.3
>95 years	22.7	29.5

Data shown as percentages.

Figure S2. *Coefficients of association between each comorbidity and diabetes mellitus, from the multiple logistic regression model, in the gout population restricted to having joint aspiration as a coded diagnosis.*

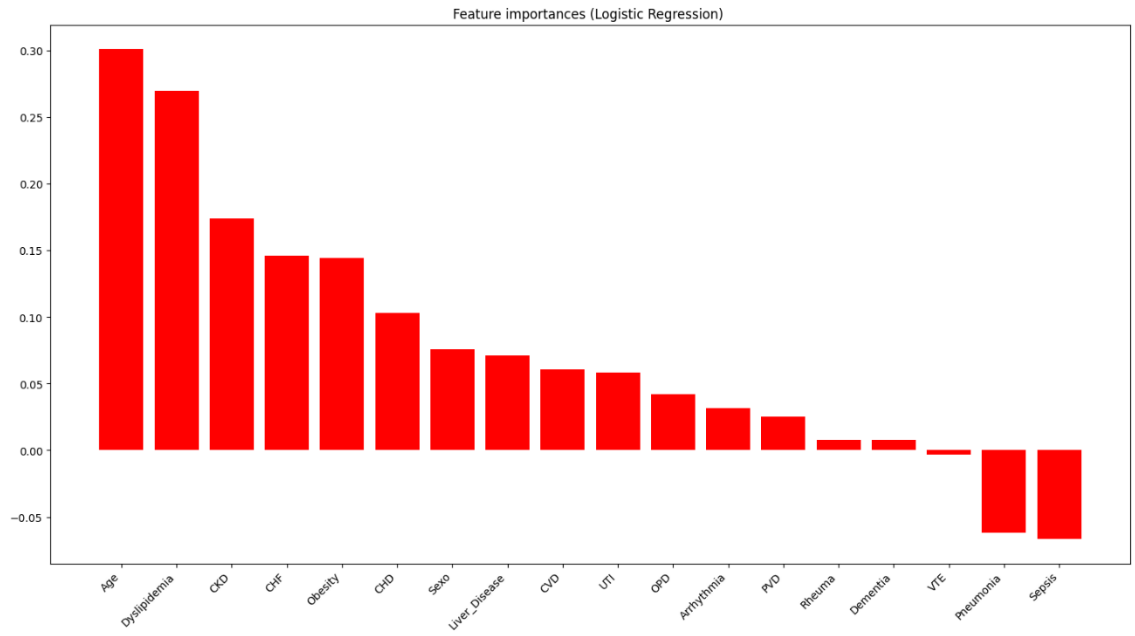

Supplement: Supplementary file 1 [file jcm-15-01925-s001.zip › jcm-4129136-supplementary.pdf]
